# Potential dsRNAs can be delivered to aquatic for defense pathogens

**DOI:** 10.3389/fbioe.2022.1066799

**Published:** 2022-11-17

**Authors:** Wenhao Nie, Xiaojiao Chen, Yueyao Tang, Nianjun Xu, Hao Zhang

**Affiliations:** Key Laboratory of Marine Biotechnology of Zhejiang Province, School of Marine Sciences, Ningbo University, Ningbo, China

**Keywords:** RNAi, aquacultur, target genes, preparation, uptake

## Abstract

The use of antibiotics to facilitate resistance to pathogens in aquatic animals is a traditional method of pathogen control that is harmful to the environment and human health. RNAi is an emerging technology in which homologous small RNA molecules target specific genes for degradation, and it has already shown success in laboratory experiments. However, further research is needed before it can be applied in aquafarms. Many laboratories inject the dsRNA into aquatic animals for RNAi, which is obviously impractical and very time consuming in aquafarms. Therefore, to enable the use of RNAi on a large scale, the methods used to prepare dsRNA need to be continuously in order to be fast and efficient. At the same time, it is necessary to consider the issue of biological safety. This review summarizes the key harmful genes associated with aquatic pathogens (viruses, bacteria, and parasites) and provides potential targets for the preparation of dsRNA; it also lists some current examples where RNAi technology is used to control aquatic species, as well as how to deliver dsRNA to the target hydrobiont.

## 1 Introduction

Aquatic animals are very important part of the contemporary food and along with other industries dominate the global economy. Currently, aquatic organisms are severely endangered, with infections by various bacterial, fungal pathogens and invasion by viruses causing a significant decline in aquatic animal populations. For example, infectious myonecrosis virus (IMNV) poses a serious threat to shrimp farming in many countries around the world, especially in Brazil (Andrade et al., 2022). Decapod iridescent virus 1 (DIV1) is an emerging virus that has posed a serious threat to crustacean farming in recent years and has caused significant economic losses (Liao et al., 2022). White spot syndrome virus (WSSV) is considered one of the most devastating diseases for shrimp farming, and there are many ways to prevent the invasion of this virus (Kumar et al., 2022). Much research has been devoted to the safety of aquatic products. For a long time, antibiotics have been used to protect aquatic animals from pathogens (Jones, 1986; Xu et al., 2021), but they have severely affected the marine ecosystem, causing eutrophication of the water and also posing a risk to humans. New ways of aquatic control are thus being sought.

The phenomenon of RNAi (RNA interference) is a molecularly mediated post-transcriptional gene-silencing mechanism, and the molecule is known as double-stranded RNA., which was discovered in plants (Napoli et al., 1990) and in *Caenorhabditis elegans* in 1998 (Andrew Fire, 1998). Since the existence of RNAi was reported, researchers began to use it to study the functions of certain genes. Also, RNAi can be used for biological control, likely aquaculture and insects. (Katoch et al., 2013). In this review, we summarize the applications of RNAi in some aquatic organisms, including studies targeting viral, bacterial, and parasitic genes. We also explore the latest pathways used to prepare RNAi and look at the future of RNAi.

## 2 Mechanisms of RNAi

RNAi is a pathway through which gene expression is downregulated, which consists of dsRNA targeting specific mRNAs inside and outside the cell for degradation (Hannon, 2002). RNAi represents an innate immune system (Reshi et al., 2014; Gong and Zhang, 2021), and as an innate immune response, when an exogenous mRNA enters a cell, it is cleaved to inhibit the replication and translation of the mRNA (Hannon, 2002). It is this property that allows RNAi technology to target certain genes for knock out and suppress viral invasion. In the initiation phase of RNAi, dsRNA is cleaved into small segments of 21–23 nucleotides by an RNase-III-like enzyme, specifically an ATP-dependent enzyme called Dicer (Hamilton and Baulcombe, 1999; Zamore et al., 2000; Bernstein et al., 2001). These small nucleotide segments of double-stranded RNA are called siRNA and have approximately 19 bp duplexes and two-base 3′-overhangs(Lingel and Izaurralde, 2004). The siRNA is then involved in the formation of the RNA-induced silencing complex (RISC). RISC is the key role in RNAi technology (Chendrimada et al., 2005; Schuster et al., 2019).

## 3 RNAi-mediated inhibition of *Pseudomonas*

*Pseudomonas plecoglossicida* is a pathogen that can cause significant harm to marine organisms (Zhang et al., 2013) and has caused serious economic losses to the marine economy. Since the isolation of *P. plecoglossicida*, its pathogenic mechanism has been extensively studied, from which relevant therapeutic targets can be discovered (Table 1). The silencing of genes in the bacterium *via* RNAi technology can significantly reduce mortality of the host, and these genes share similar functions, namely, toxicity, adhesion, and flagellar movement.

**TABLE 1:** The genes of *Pseudomonas* can be inhibited by RNAi.

Gene	Function	References
*clpV*	Reducing mortality	Sun et al. (2019); Tang et al. (2019c); Wang et al. (2019a); Qi et al.,(2022)
*fliA*	Reducing mortality	
*L321_RS13075*	Reducing mortality	Wang et al. (2020)
*secY*	Reducing mortality	Zhang et al. (2018a)
*dksA*	Flagellum and ribosome assembly	Qi et al. (2019)
*impB*	Reducing lethality and stimulating immunity	Tang et al. (2019a)
*L321_RS1911*	Reducing lethality and stimulating immunity	Zhang et al. (2018a); Qi et al. (2019); Tang et al. (2019a); Tang et al. (2019b); Zhang et al. (2019a); Liu et al. (2020b); Hu et al. (2021); Jiao et al. (2021); Tang et al. (2022)
*L321_23611*	Reducing lethality and stimulating immunity	
*L321_20267*	Reducing lethality and stimulating immunity	
*fliG*	Reducing lethality and stimulating immunity	
*TonB*	Reducing lethality and stimulating immunity	Hu et al. (2021)
*cspA1*	Reducing lethality and stimulating immunity	Luo et al. (2019)
*RK21_RS10315*	Reducing lethality and stimulating immunity	He et al. (2022)
*ExbB*	Reducing lethality and stimulating immunity	Huang et al. (2019b)
*L321_RS15240*	Influence on host metabolism	Huang et al. (2019b)
*fusA*	Ferrous oxygen reducing Sulfur protein transport	Ilari et al. (2016); He et al. (2021); Huang et al. (2021)
*htpG*	Biofilm formation, adhesion and toxicity	
*RpoE*	Biofilm formation, adhesion and toxicity	
*ZnuABC*	Blocking the absorption of elements	

Trace elements such as Mn and Zn are necessary for bacterial growth, and these elements are only available through the host, which uses a pathway to prevent bacterial uptake of the elements (Kehl-Fie and Skaar, 2010; Hood and Skaar, 2012). Zn is a very important element that plays a catalytic role in proteins and is also able to maintain protein functions and partial bacterial toxicity (Hantke, 2005). Many bacteria transport Zn *via* the ZnuABC transporter (Ilari et al., 2016). It was found that ZnuC is an important protein for Zn uptake in *Pseudomonas aeruginosa* (Huang et al., 2021). Similarly, the authors found that silencing the *znuA* gene in this strain could achieve an 89.2% reduction of deaths and that RNAi-treated strains also induced antibody production in grouper (He et al., 2021). We have summarized some genes that we expect to enhance host defense through RNAi technology (Table 1).

## 4 RNAi-mediated inhibition of *Aeromonas hydrophila*

*Aeromonas hydrophila* is a species of *Aeromonas* capable of causing significant harm to both aquatic organisms and humans, impacting the aquatic industry worldwide (Sha et al., 2002; Sabili et al., 2015). Until the mechanisms of *A. hydrophila* virulence were understood, antibiotics were used to control related diseases (Samir et al., 2017). With the introduction of a large number of antibiotics, *A. hydrophila* has developed drug resistance (Mao et al., 2020). People found silencing *LuxR* by using RNAi technology can partially restore the susceptibility of *A. hydrophila* to antibiotics (Chang et al., 2010). Therefore, inhibition of this strain with RNAi is a promising approach. Many genes have important roles in *A. hydrophila* biofilm formation, bacterial motility, and virulence.

The escape of bacteria in macrophages is necessary for a rapid infection process (Qin et al., 2014). In an *in vitro* invasion assay of *A. hydrophila*, *flgE* was found to be an important gene for flagellogenesis and key to the infestation of macrophages (Qin et al., 2014). Further, *acuC*-RNAi strains were able to reduce mortality in zebrafish (Jiang et al., 2017), which also resulted the expression of *hlyA* (a key virulence gene of *A. hydrophila*) decreased (Qian et al., 1995), demonstrating that acuC is an important regulatory protein affecting the survival and pathogenicity of *A. hydrophila*. Moreover, icmF is thought to be an ATPase capable of stimulating the T6SS secretome of *A. hydrophila* (Ma et al., 2012). The maximum efficiency of knockdown for *icmF* using RNAi technology can reach 94.42%, resulting in a decrease in the survival of *A. hydrophila* from 92.3% to 20.58%, as well as a significant reduction in the probability of escape from macrophages(Wang S. et al., 2019).

Reactive oxygen species (ROS) are an important way for host macrophages to mitigate bacterial invasion (Grayfer et al., 2014). Zhang et al. (Zhang M. et al., 2018) found that katG facilitates the removal of host H_2_O_2_ by *A. hydrophila* and aids in its survival in macrophages and that RNAi-*katG* could reduce immune escape and fish mortality by 85%; the authors also found that *sodA*-RNAi and *sodB*-RNAi were able to reduce *A. hydrophila* survival in fish macrophages by 91.8% and 74.9% and reduce immune evasion by 32% and 92%, respectively, in addition to restoring ROS content in some macrophages and enhancing host immunity (Zhang M. et al., 2019). These genes are summarized. (Table 2).

**TABLE 2:** The genes of *Aeromonas hydrophila* can be inhibited by RNAi.

Gene	Function	References
*LuxR*	Biofilm formation, adhesion and toxicity	Qian et al. (1995); Zeng and Xie, (2011); Bontemps-Gallo et al. (2019); Zhang et al. (2020)
*EnvZ*	Biofilm formation, adhesion and toxicity	
*OmpR*	Biofilm formation, adhesion and toxicity	Qian et al. (1995)
*hlyA*	Biofilm formation, adhesion and toxicity	Lin et al. (2017), Huang et al. (2015b); Lin et al. (2017), Huang et al. (2015b)
*RbsR*	Adhesion	
*MinD*	Adhesion	Qin et al. (2014)
*flgE*	Macrophage infection	Wang et al. (2019b)
*icmF*	Escaping macrophages	Zhang et al. (2018b); Zhang et al. (2019b)
*katG*	Reactive oxygen species	
*sodA*	Reactive oxygen species	Zhang et al. (2019b)
*sodB*	Reactive oxygen species	Jiang et al. (2017)
*acuC*	Critical regulation of survival and pathogenicity and reducing mortality	

## 5 RNAi to target *Vibrio alginolyticus*

*Vibrio alginolyticus* is capable of harming coral polyps, aquatic organisms (Zhenyu et al., 2013; Huang et al., 2019b), and crustaceans. Oxidative phosphorylation is an important pathway for aerobic growth and energy acquisition, suggesting that key oxidative phosphorylation proteins are associated with bacterial adherence. However, silencing the relevant oxidative phosphorylation protein-encoding genes *via* RNAi technology can result in reduced bacterial adherence and cytochrome C oxidase activity. The adherence in *V. alginolyticus* is critical in the early stages of pathogenesis, and there have been many studies on targeting adhesion genes using RNAi for *Vibrio* lysozyme (table below), including some tricarboxylic acid cycle genes (Huang et al., 2016b). These proteins could be potential targets for the RNAi-mediated control of aquatic pathogens. Zhang (Liu et al., 2012) found that luxT promotes the transcription of luxO and luxR, luxO can regulate MviN (an extracellular protein that produces toxicity) (Cao et al., 2010), and luxO promotes the secretion of extracellular substances and the formation of iron carriers in *V. alginolyticus* (Wang et al., 2007). The pep protein (a protein required for *Vibrio* lysis motility) is also regulated by luxO (Cao et al., 2011), and luxR regulates extracellular alkaline serine protease A and reduces the production of extracellular sugars and motility in *V. alginolyticus* (Rui et al., 2008). LuxR and AphA are two of the most important molecules involved in sensing the *Vibrio* lysogenicus population. One related study found that the *AphB* (Gao et al., 2017) gene can positively regulate the expression of luxR and the toxin asp (alkaline serine protease), which is expected to reduce *Vibrio* lysis by repressing AphB, whereas AphA can negatively regulate the asp toxin through luxR (Gu et al., 2016). Further, luxO-luxR can regulate asp production (Rui et al., 2009). *ValR*, a gene homologous to *luxR*, affects both cell membrane formation and bacterial motility by regulating flagellar synthesis (Chang et al., 2010). DctP, a protein-transporting subunit, was found to regulate the expression of 22 genes involved in the pathogenesis of *Vibrio* lysogenicus, without inducing any morphological changes, but with significantly reduced adherence and virulence (Zhang et al., 2022). VqsA (Gao et al., 2018) is a transcription factor that functions in Type VI secretion systems. AcfA is a factor required for *Vibrio* infection, and in acfA-deficient strains in which *DctP* (Zhang et al., 2022) and *deoD* transcript levels were found to be increased, *pepD*, *arA*, *fla*, and *ompA* genes are repressed (Cai et al., 2018). Both TonB systems are important for virulence in *Vibrio* lysogenicus, and the absence of TonB diminishes this virulence (Wang et al., 2008). A previous team used pulsed-field gel electrophoresis to isolate a number of *Vibrio* pathogenesis-related genes for subsequent RNAi technology (Ren et al., 2013). Huang et al. (Huang et al., 2015a; Huang et al., 2016a) used high-throughput sequencing to find a number of non-coding RNA molecules that they believe play a key role in *Vibrio* infection of the host. Srvg17985, a small RNA that regulates various aspects of stress balance in *Vibrio*, is involved in adapting to environmental stress, and is expected to be a new target (Deng et al., 2019). Vvrr1 (a non-coding RNA) and pykF interact with each other and are involved in the mechanism of virulence in *V. alginolyticus* (Zuo et al., 2019). Qrr (Liu H. et al., 2020) is a non-coding small molecule RNA that activates luxR and inhibits aphA.

Micronutrient uptake is necessary for growth and physiological processes in bacteria (Kehl-Fie and Skaar, 2010; Hood and Skaar, 2012). OmpU is a pore protein located on the surface of *V. alginolyticus* that regulates Fe uptake, and physiological growth is compromised in strains lacking this protein (Lv et al., 2020). Branched-chain amino acid metabolism is also capable of influencing bacterial physiological activity. Deng et al. (Deng et al., 2017) detected 311 upregulated genes and 251 downregulated genes in nitrogen source culture, which provides a foundation for subsequent RNAi applications. Genes which have mentioned are summarized. (Table 3).

**TABLE 3:** The genes of *Vibrio alginolyticus* can be inhibited by RNAi.

Gene	Function	References
*FlrA,B,C*	Biofilm formation, adhesion and toxicity	Liu et al. (2011); Luo et al. (2016); Huang et al. (2017); Liu et al. (2017); Guo et al. (2018); Huang et al. (2018); Zhang et al. (2022) Yang et al. (2018) Zuo et al. (2019)
*rstA,B*	Biofilm formation, adhesion and toxicity	
*mcp*	Biofilm formation, adhesion and toxicity	
*SecA,D,F*	Biofilm formation, adhesion and toxicity	Guo et al. (2018)
*Opp*	Biofilm formation, adhesion and toxicity	Cai et al. (2018)
*DctP*	Biofilm formation, adhesion and toxicity	Gu et al. (2019); Huang et al. (2019a)
*PppA*	Biofilm formation, adhesion and toxicity	Tian et al. (2008)
*Vvrr1*	Biofilm formation, adhesion and toxicity	Liu et al., (2012)
*AcfA*	Biofilm formation, adhesion and toxicity	Gao et al. (2017)
*RpoS,E,X*	Propagation of strains and virulence	Gao et al. (2018)
*LuxS*	Flagellum assembly	Liu et al. (2020a)
*LuxT,R*	Factors of regulation	Cao et al., (2010)
*AphB*	Factors of regulation	Gu et al., (2016)
*VqsA*	Factors of regulation	Chang et al., (2010)
*Qrr*	Factors of regulation	Zhou et al., (2013)
*luxO*	Factors of regulation and iron carrier formation	Pang et al., (2018)
*AphA*	Group-sensing molecule	
*valR*	Regulation of flagellar synthesis	Chen et al., (2019)
*VscO*	T3SS secretion system	Lv et al., (2020)
*hopP*	T3SS secretion system	Deng et al., (2019); Zhou et al. (2013)
*sodB*	Antioxidant and toxicity	
*OmpU*	Regulation of Fe intake	
*srvg17985*	Pressure balance	

## 6 Viruses and RNAi

RNAi-mediated long dsRNA or siRNA causes cellular resistance to foreign nucleic acid invasion and is a natural mechanism prevalent in many species (Keene et al., 2005; Wang et al., 2006; Ding and Voinnet, 2007). Aquatic animals can be infected by many types of viruses, and one virus can carry multiple disease-causing genes (Table 4). The injection of these specific viral sequences into shrimp enables the shrimp to produce RNAi to resist the virus. YHV (yellow head virus), is a positive sense, single-stranded RNA virus; WSSV, white spot syndrome virus, is a DNA virus comprised of double-stranded circular DNA, and we list some evidence for RNAi silencing of key viral genes. It has been demonstrated that YHV-specific dsRNA introduced into spotted shrimp can effectively inhibit the replication of YHV (Tirasophon et al., 2005; Yodmuang et al., 2006; Tirasophon et al., 2007).

**TABLE 4:** The genes of viruses can be defended by RNAi.

Virus	Gene	Host	Delivery	Production	References
YHV	*gp116,gp64*	*P. monodon*	Transfection	Transcribed dsRNA	Tirasophon et al. (2005)
	*RdRp*	*P. monodon*	Transfection	Transcribed dsRNA	Thedcharoen et al. (2020a)
	*RdRp*	*L. vannamei*	Injection	Bacterial expressed dsRNA	Saksmerprome et al. (2009)
	*RdRp*	*L. vannamei*	Oral	Microalgal expressed dsRNA	Charoonnart et al. (2019)
	*rr2*	*L. vannamei*	Injection	Bacterial expressed dsRNA	Chaimongkon et al. (2020)
	*EEA1*	*P. monodon*	Injection	Bacterial expressed dsRNA	Posiri et al. (2019)
WSSV	*Vp28*	*L. vannamei*	Oral	Synthesized	Ramos-Carreño et al. (2021)
	*Vp28*	*L. vannamei*	Injection	Transcribed dsRNA	Nilsen et al. (2017)
		*M. japonicus*	Injection	Synthesized	Xu et al. (2007)
	*Vp37*	*L. vannamei*	Injection	Synthesized	Weerachatyanukul et al. (2021)
	*rr2*	*L. vannamei*	Injection	Bacterial expressed dsRNA	Chaimongkon et al. (2020)
	*V9*	*P. monodon M. japonicus*	Injection	Synthesized	Alenton et al. (2016)
	*V26*	*L. vannamei*	Injection	Transcribed dsRNA	Mejía-Ruíz et al. (2011)
GAV	*β-actin*	*P. monodon*	Oral	Bacterial expressed dsRNA	Sellars et al. (2011)

## 7 Parasites and RNAi

Salmon farming has been largely affect by *Lepeophtheirus salmonis* (salmon louse) (Brooker et al., 2018). AGD (Amoebic gill disease) is a parasitic disease of salmonids (Crosbie et al., 2012). The use of large amounts of anti-parasitic drugs seems to alleviate the development of the association disease (Carmichael et al., 2013). But as we mentioned before, drug abuse can put our health at risk. We use RNAi to target certain pathway genes, which can also prevent parasitism. For example, inhibition of the titin synthesis pathway in this parasite is a very effective way of targeting it (Liu et al., 2019). We list some examples of key genes in aquatic parasites that have been knocked out using RNAi technology (Table 5).

**TABLE 5:** The genes of some parasites in hosts can be defended by RNAi.

Parasite	Gene	Host	Delivery	Production	References
*Lepeophtheirus salmonis*	*LsCHS1*	Salmon	Incubation	Synthesized	Braden et al. (2020)
	*LsCHS2*	Salmon	Incubation	Synthesized	Braden et al. (2020)
	*LsGFAT*	Salmon	Incubation	Synthesized	Braden et al. (2020)
	*LsUAP*	Salmon	Incubation	Synthesized	Braden et al. (2020)
	*LsAGM*	Salmon	Incubation	Synthesized	Braden et al. (2020)
	*LsCDA4557*	Salmon	Incubation	Synthesized	Braden et al. (2020)
	*LsCDA5169*	Salmon	Incubation	Synthesized	Braden et al. (2020)
	*LsCDA5956*	Salmon	Incubation	Synthesized	Braden et al. (2020)
	*MLSWP1,2*	Salmon	Injection	Synthesized	Borchel and Nilsen, (2018)
	*Na+/K ± ATPase*	Salmon	Incubation	Synthesized	Komisarczuk et al. (2018)
Neoparamoeba pemaquidensis	*β-actin*	Salmon	Incubation	Bacterial expressed dsRNA	Lima et al. (2014)
	*EF1-α*				

## 8 Preparation and uptake of RNAi

Many RNAi molecules can now be injected in the laboratory to control the death of aquatic animals, but this is a time-consuming and impractical approach for aquaculture farms. The uptake of RNAi molecules in cells is hampered by properties, such as the molecular length and volume, charge, and nuclease degradation (Gupta et al., 2019; Sajid et al., 2020). Therefore, to apply RNAi in aquaculture farms, two conditions must be met as follows: 1) more dsRNA production (including the preparation of dsRNA) and 2) the stable presence of dsRNA in aquatic organisms (including ingestion of dsRNA).

### 8.1 Preparation

#### 8.1.1 Expressing in bacteria

The use of fed-batch fermentation was able to increase the growth of the bacteria and increase the nucleic acids. The fermentation of *E. coli* HT115 (DE3) was able to increase the growth of the bacteria, increase the amount of nucleic acid and then increase the level of dsRNA molecular, and maximize the expression function of *E. coli* HT115 (DE3) (Papić et al., 2018). Moreover, in *E. coli*, p19, an siRNA binding protein, stabilizes the siRNA produced by this bacterium and increases the amount transferred to siRNA (Huang and Lieberman, 2013).

*Yarrowia lipolytica* is also a harmless bacterium. Álvarez-Sánchez et al. (Álvarez-Sánchez et al., 2018) used *Y. lipolytica* to produce dsRNA to help *L. vannamei* resist WSSV, and when *Y. lipolytica* produced hairpin RNA against WSSV-orf89, injection of the extracted RNA into the muscle of shrimp improved survival after WSSV infection by 25%.

*Lactobacillus plantarum* is a plant-derived lactic acid bacterium that is harmless to humans. Thammasorn et al. (Thammasorn et al., 2017) modified *L. plantarum* to produce hairpin RNA by transferring genes targeting YHV specific to this probiotic and were able to enhance resistance to YHV in shrimp consuming the probiotic while retaining the original function of the probiotic, which is to inhibit the development of some diseases. Another probiotic, W2, was found to be non-toxic, containing an active bacteriostatic component, and was able to antagonize seven strains of aquatic pathogens, while also promoting shrimp growth (Wei et al., 2022). W2 appears to be equally useful for RNAi control in the same manner as *L. plantarum*. Dekham et al. (Dekham et al., 2022) transferred hpRNA targeting WSSV-vp28 into *L. plantarum* and *Lactococcus lactis via* pWH1520-VP28 and found that both probiotics were able to protect the host against the virus, with *L. lactis* not only reducing shrimp mortality due to WSSV but also stimulating RNAi and activating the innate immune system of the shrimp.

#### 8.1.2 Reorganization of the particles

Sarathi et al., (2008) fed *Penaeus monodon* inactivated bacteria specifically expressing dsRNA from WSSV vp28 and pelleted feed covered with vp28 dsRNA-chitosan composite nanoparticles and invaded P. monodon by WSSV. Romo-Quiñonez et al. (Romo et al., 2020) were able to enhance the antiviral potential of *Litopenaeus vannamei* after using a silver nanoparticle, Argovit4, administered as feed. Sinnuengnong et al., (2018) co-expressed virus-like particles of pstDNV with dsRNA molecules of YHV-Pro to deliver dsRNA using viral proteins, which was also very effective. Thedcharoen et al., (2020b) used the pLVX-AcGFP1-N1 vector with a gene encoding long hairpin RNA, namely pLVX-lhRdRp, introduced under control of the CMV promoter, to inhibit the RdRp (RNA-dependent RNA polymerase) of YHV with significant effects.

#### 8.1.3 Small creatures

Artemia, a common feed for marine organisms, is able to enrich proteins and other molecules in the gut (Subhadra et al., 2010). Feeding *P. monodon* with LSNV-enriched dsRNA artemia can effectively inhibit LSNV infection (Thammasorn et al., 2013).

Microalgae possess essential nutrients for aquatic organisms, and many natural antimicrobial substances have become a promising new green and environmentally friendly platform (Charoonnart et al., 2018; Fayyaz et al., 2020). *Chlamydomonas reinhardtii* is a distinct group of microalgae in which exogenous genes can be recombined into nuclear DNA or chloroplast DNA to express recombinant proteins (Rosales-Mendoza et al., 2012; Shamriz and Ofoghi, 2016). Somchai et al. (Somchai et al., 2016) transferred dsRNA targeting YHV-RdRp into the nuclear genome of *C. reinhardtii* to produce hairpin RNA. Feeding this to larval shrimp increased their survival by 22% after YHV infection. However, eukaryotic cell nuclei have mechanisms associated with RNAi that target dsRNA for degradation, which are lacking in chloroplasts (Boynton et al., 1988). In chloroplasts, dsRNA can accumulate and produce large amounts of dsRNA. At the same time, gene transformation in chloroplasts is not affected by off-target effects, mutations, and antibiotic screening (Doron et al., 2016). Thus, chloroplast transformation to produce dsRNA is more advantageous than nuclear transformation. Charoonnart et al., (2019) transferred dsRNA targeting YHV-RdRp into chloroplasts of *C. reinhardtii*, and 8 days after YHV infection, the survival of shrimp consuming dsRNA-expressing microalgae increased by 34% compared to that in controls, although the microalgae produced less dsRNA than that with nuclear transformation. Purton et al. (Kiataramgul et al., 2020) used WSSV-vp28 gene to integrate into *C. reinhardtii* which is a chloroplast of cell wall-deficient, and the *C. reinhardtii* was fed to shrimp, and the survival rate of shrimp was 87% in the presence of WSSV invasion. Pham et al., (2021) transferred the vp28 protein of WSSV into the nucleus of *C. reinhardtii* cells, and after feeding, the shrimp developed an immune response against WSSV. These results indicate that *C. reinhardtii* is a very promising oral substance that could be used to protect aquatic animals from pathogens.

### 8.2 Oral delivery is very common

Rattanarojpong et al., (2016) transferred dsRNA targeting the *rr2* gene into a recombinant baculovirus expressing vp28 and then injected it into shrimp. Shrimp mortality was greatly reduced and WSSV infection was also prevented. Weerachatyanukul et al., (2021) co-packaged genes for silencing vp28 and vp37 into a virus like particle-IHHNV and injected this particle into shrimp, which not only improved survival but also stimulated the immune response.

The most promising method of RNAi delivery in aquatic waters is oral. Pathogens such as microorganisms and viruses can overcome host immunity and cellular barriers that facilitate the delivery of RNAi molecules (Abo-Al-Ela, 2020). Thammasorn et al. (Ongvarrasopone and Panyim, 2007; Thammasorn et al., 2015) used *Escherichia coli* to express dsRNA targeting the vp28 and WSSV051 structural protein-encoding *genes* of WSSV and used oral delivery of dsRNA to shrimp, which was able to reduce the risk of WSSV infection and mortality in shrimp, and this method was less costly than traditional transcriptional techniques.

They found that the numbers of surviving inactivated bacteria consumed were higher than those in shrimp that were orally fed nanoparticles, suggesting that the oral delivery of dsRNA-expressing bacteria improves host survival, as illustrated in another study (Leigh et al., 2015).

## 9 Outlook

Aquaculture is becoming increasingly important and a major support for the world economy. At the same time, there are many pathogens in aquaculture that are constantly attacking aquatic organisms. People began to use antibiotics in large quantities to address this (Monahan et al., 2021), which has had a considerable impact on the environment and human health. With the continuous research on and maturity of RNAi technology, researchers have shifted their attention from antibiotics to RNAi for the control of aquatic aquaculture. This paper highlights the potential dsRNA target genes in aquatic pathogens that can reduce pathogen infection and improve the survival of aquatic host animals after silencing and also introduces species used as feed for oral-based dsRNA, such as *E. coli* and microalgae. An increasing number of studies has shown that the use of *E. coli* is a very inexpensive way to synthesize dsRNA, and *E. coli* is constantly being modified to obtain high yields (Hashiro and Yasueda, 2022).

However, the long-term effects of the regular use of *E. coli* cells in shrimp feed on animals and the environment have yet to be studied. Therefore, finding alternative dsRNA production and delivery systems that can be used safely in shrimp farms is a key challenge for future applications of this technology. *C. reinhardtii* is considered a very safe organism (Enzing et al., 2014) that has great appeal as a cell factory. Probiotics are also very promising as oral feed for delivering dsRNA, while enhancing host immunity.

At the same time, to be able to apply RNAi technology in farms, we have to consider the cost and safety. Since dsRNA molecules are constantly degraded, a large amount of dsRNA is needed for the organisms, and ways to increase the yield while controlling the cost is a problem that needs to be solved (Papić et al., 2018; Huang and Lieberman, 2013). The use of RNAi technology for aquatic control needs to be improved and the threat to biodiversity needs to be further explored. The continuous development of RNAi molecule production and delivery methods targeting pathogen genes to reduce off-target effects and enhance host resistance remains a direction to be pursued.
